# All That Swells Is Not Allergy: Unmasking Rhino-Orbital-Cerebral Mucormycosis in Diabetic Ketoacidosis

**DOI:** 10.7759/cureus.107978

**Published:** 2026-04-29

**Authors:** Carlos Fagundo, Catherine Xu, Hazem Abugrara

**Affiliations:** 1 Internal Medicine, BayCare Health System, Tampa, USA; 2 Hospital Medicine, BayCare Health System, Tampa, USA

**Keywords:** diabetic ketoacidosis (dka), infectious disease medicine, invasive fungal infections, rhino-orbital mucormycosis, type 2 diabetes

## Abstract

Rhino-orbital-cerebral mucormycosis (ROCM) is the most common manifestation of mucormycosis in uncontrolled diabetes and diabetic ketoacidosis (DKA). This infection is extremely aggressive, invading blood vessels and spreading quickly from the sinuses to the orbit and brain. We present a case of a 57-year-old male with poorly controlled type 2 diabetes who came to the hospital with facial swelling, blurry vision, and severe DKA. Imaging revealed orbital cellulitis, sinusitis with gas formation, and cavernous sinus thrombosis. He underwent an urgent lateral canthotomy and surgical debridement. Pathology confirmed invasive mucormycosis, and he was started on liposomal amphotericin B. Despite aggressive treatment, the infection progressed to the brain, causing multiple strokes. The patient’s condition continued to decline, and he was eventually transitioned to hospice care. This case emphasizes the importance of early recognition of mucormycosis in high-risk patients to prompt urgent evaluation. Because the infection spreads so rapidly and antifungal medications penetrate the brain poorly, early surgical intervention and systemic therapy are vital.

## Introduction

Mucormycosis is a rare but highly aggressive fungal infection caused by molds of the order *Mucorales*, with *Rhizopus* species being the most common pathogen [1]. Although the spores are ubiquitous in the environment, clinical infection is infrequent and typically occurs in immunocompromised hosts [1,2]. Uncontrolled diabetes mellitus, particularly during diabetic ketoacidosis (DKA), remains the leading risk factor that creates a uniquely permissive environment for fungal proliferation [3-5].

The most frequent clinical manifestation is rhino-orbital-cerebral mucormycosis (ROCM), which begins in the paranasal sinuses and rapidly progresses to involve the orbit and central nervous system (CNS) [3,6,7]. Early symptoms of ROCM can be nonspecific, including facial pain, swelling, ophthalmoplegia, or vision changes, which may mimic bacterial sinusitis or allergic reactions [6,8,9]. Without prompt recognition and treatment, ROCM can result in vascular thrombosis, tissue necrosis, and cerebral infarction [3,10]. Despite aggressive antifungal and surgical management, mortality approaches 100% once the CNS is involved [3,11].

Diagnosis relies on a high index of clinical suspicion in at-risk patients. Initial imaging with contrast-enhanced CT or MRI is critical to assess for sinus opacification, orbital extension, or vascular complications such as cavernous sinus thrombosis [8,12]. Definitive diagnosis requires histopathologic identification of broad, non-septate hyphae, often supported by fungal culture [13-15]. However, traditional fungal cultures are slow and may be negative in up to 50% of cases [13,15,16].

We present a case of ROCM in a 57-year-old male with poorly controlled diabetes mellitus and DKA whose initial symptoms were mistakenly attributed to an allergic reaction. The atypical presentation and findings in this case highlight the importance of early clinical suspicion and intervention.

## Case presentation

A 57-year-old African American male with a history of hypertension and poorly controlled type 2 diabetes mellitus (glycosylated hemoglobin (HbA1c) = 13%) initially presented to his primary care provider complaining of upper respiratory tract and sinusitis symptoms for which he was prescribed azithromycin and albuterol. After two weeks, the patient reported a lack of improvement and was sent to a hospital-satellite emergency department by his primary care physician, complaining of left-sided facial swelling. His symptoms were attributed to an allergic reaction to antibiotics for which he was given Benadryl and steroids, and subsequently discharged.

The following day, he returned with progressive facial swelling, blurry vision in the left eye, and altered mental status. Laboratory workup revealed leukocytosis (WBC = 30 x10^9/L), and sodium level of 111 mmol/L, potassium level of 7.9 mmol/L, glucose level of 1,128 mg/dL, creatinine at 2.1 mg/dL, anion gap of 27, lactic acid at 5.3 mmol/L, procalcitonin at 2.39 ng/mL, and arterial blood gas (ABG) showing a pH of 7.23, partial pressure of carbon dioxide (pCO_2_) of 19 mm/hg, partial pressure of oxygen (pO_2_) of 55 mm/hg, and bicarbonate (HCO3) at 7.9 mm/hg (Table 1). A CT of the orbits without contrast showed left-sided sinusitis with possible dehiscence of the lateral wall of one of the ethmoid cells and inflammation extending into the soft tissues of the nose and into the left orbit. Induration of the fat in the left orbit is consistent with cellulitis. Given his persistent pain, vision loss, and intraocular pressure of 29 mm/hg, an emergent lateral canthotomy was performed. The patient was started on intravenous insulin, fluids, and broad-spectrum antibiotics and was subsequently airlifted to our emergency department for further evaluation and management.

**Table 1:** Laboratory values. Patient's lab values on admission to the hospital, with associated interpretation. ABG: arterial blood gas; pCO₂: partial pressure of carbon dioxide; pO₂: partial pressure of oxygen; HCO₃⁻: bicarbonate.

Lab test	Value	Units	Reference range	Interpretation
White blood cells	30	×10⁹/L	4-11	High
Sodium (Na⁺)	111	mmol/L	135-145	Low
Potassium (K⁺)	7.9	mmol/L	3.5-5.0	High
Glucose	1128	mg/dL	70-100	High
Creatinine	2.1	mg/dL	0.6-1.3	High
Anion gap	27	mmol/L	8-12	High
Lactic acid	5.3	mmol/L	0.5-2.0	High
Procalcitonin	2.39	ng/mL	<0.1	High
pH (ABG)	7.23	-	7.35-7.45	Low
pCO₂	19	mmHg	35-45	Low
pO₂	55	mmHg	80-100	Low
HCO₃⁻	7.9	mmol/L	22-26	Low

A repeat maxillofacial sinus CT at our facility showed left orbital cellulitis and extensive paranasal sinus disease with increased soft tissue gas. Examination by ophthalmology showed marked proptosis of the left eye, +3 conjunctival chemosis, and denuded periorbital skin with blood‐tinged nasal discharge. The left pupil was fixed and dilated with severely restricted extraocular motility. Indirect ophthalmoscopy showed an ischemic-appearing optic nerve and attenuated retinal vessels without papilledema or diabetic retinopathy. The cornea, anterior chamber, and vitreous were unremarkable. Flexible nasal endoscopy by otolaryngology (ENT) showed a normal right nasal cavity.

**Figure 1:**
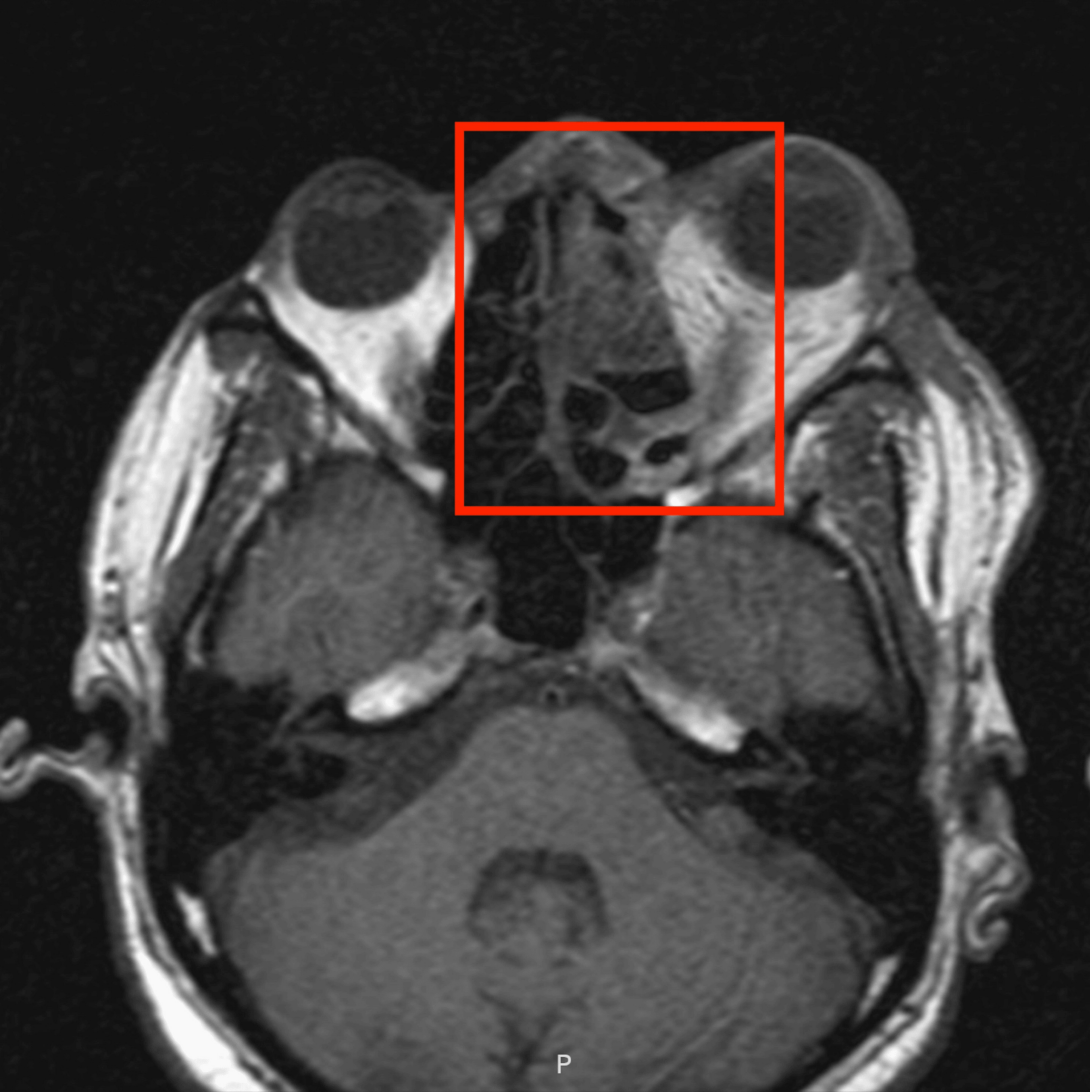
Axial contrast-enhanced MRI demonstrates non-enhancement of the left cavernous sinus, with associated left orbital cellulitis, proptosis, and the left lateral rectus muscle. A red box has been placed over the area of concern.

The left cavity was obscured by marked mucosal edema and purulence; anatomic landmarks were not visualized. Erythematous serosanguinous drainage was present, but no frank necrosis or thrombosis was observed (Figure 1). Examination of the oropharynx was limited by trismus. Externally, the left mid- and upper face appeared diffusely swollen from the periorbital region across the nasal sidewall and cheek to the mandibular border, consistent with progressive sino-facial cellulitis. No cervical lymphadenopathy or masses were palpable.

Repeat imaging with a non-contrast maxillofacial CT scan demonstrated complete opacification of all paranasal sinuses, intraorbital gas, and marked medial orbital soft tissue swelling. An MRI of the brain and orbits with and without contrast confirmed diffuse left orbital cellulitis with proptosis and revealed a lack of enhancement of the left cavernous sinus, concerning for cavernous sinus thrombosis (CST) (Figure 1).

**Figure 2:**
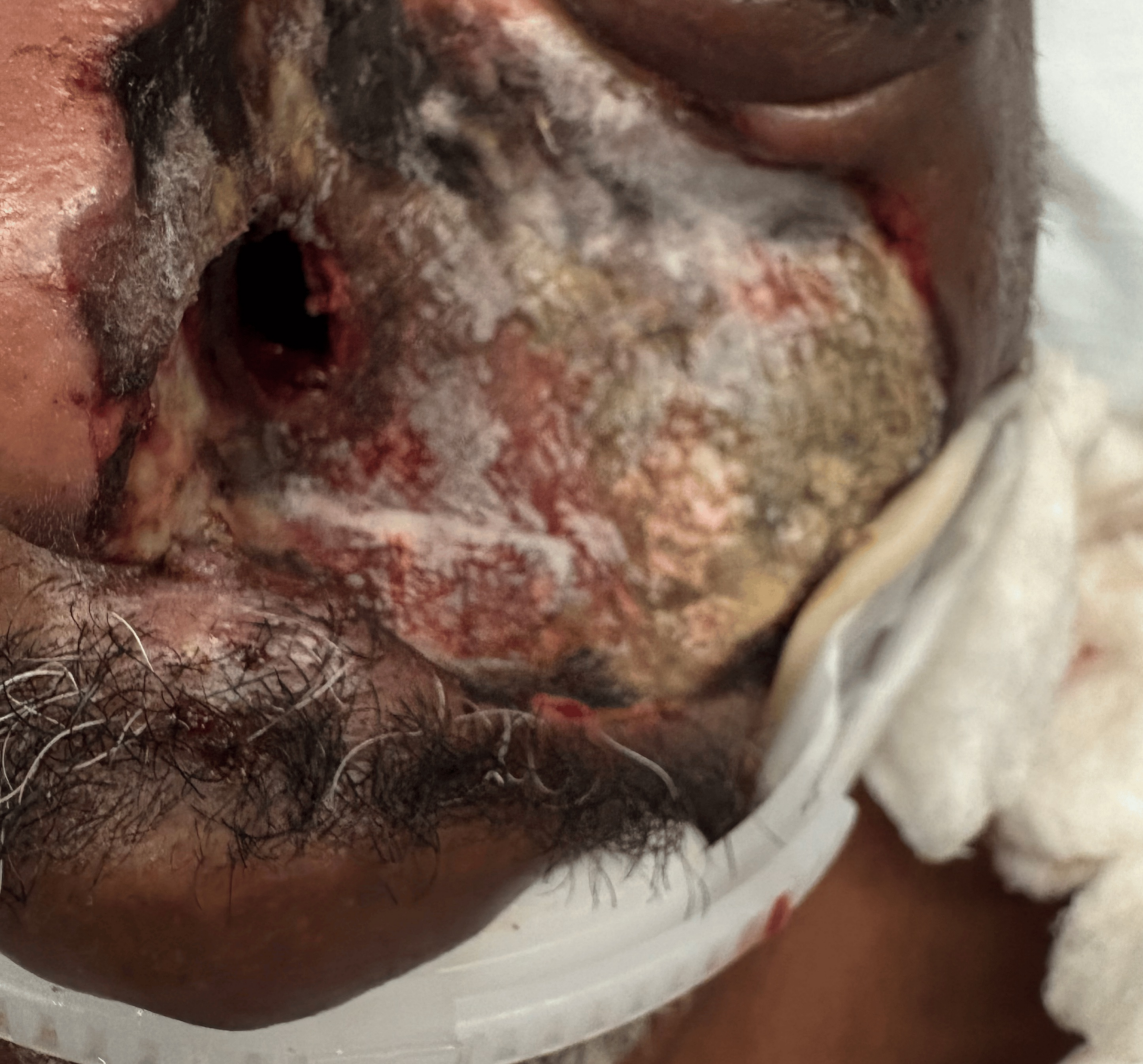
Soft tissue from the periorbital to the cheek area after debridement.

The patient underwent left endoscopic sinus surgery with debridement by ENT. Intraoperative findings revealed black necrotic mucosa filling the left maxillary, ethmoid, sphenoid, and frontal sinuses, with similar though less extensive disease on the right. Externally, devitalized soft tissue from the periorbital to the cheek was sharply excised to bleeding margins, and all specimens were sent for histopathologic analysis and culture (Figure 2).

A postoperative MRI demonstrated rapid disease progression, with multifocal restricted diffusion lesions in the left middle cerebral artery (MCA) and anterior cerebral artery (ACA) watershed territories, pons, and cerebellum, reflecting ongoing vascular invasion and early cerebral infarction. CT angiography of the head and neck demonstrated tapering of the left cervical internal carotid artery and high-grade stenosis at the cavernous-supraclinoid junction, correlating with the watershed infarcts and confirming continued hematogenous spread, despite surgical debridement and initiation of liposomal amphotericin B.

Despite initiation of high-dose liposomal amphotericin B and repeated surgical debridement, the infection progressed (Figure 2). Given extensive cerebral involvement and poor neurologic prognosis, the decision was made to transition the patient to hospice care.

## Discussion

ROCM remains one of the most aggressive and life-threatening fungal infections, particularly in patients with uncontrolled diabetes or DKA. The European Organization for Research and Treatment of Cancer and the Mycoses Study Group Education and Research Consortium (EORTC/MSGERC) have established definitions for diagnosing invasive mold infections such as mucormycosis [2]. Diagnosis is categorized as proven, probable, or possible based on a combination of host factors, clinical features, radiologic findings, and microbiologic or histopathologic evidence.

Possible disease is considered when only host factors and clinical presentation are present, without radiologic or microbiologic confirmation. The diagnosis of probable mucormycosis is made when a high-risk patient, such as a patient with uncontrolled diabetes or DKA, develops a compatible clinical syndrome such as unilateral sinusitis with facial swelling or ophthalmoplegia, and imaging demonstrates hallmark angio-invasive findings. Imaging findings suggestive of mucormycosis include unilateral sinus opacification with bony erosion, intra-orbital gas, or CST [7-9]. Proven mucormycosis is confirmed when tissue from a sterile site shows characteristic broad, ribbon-like, pauci-septate hyphae, or when *Mucorales* species are isolated through culture or PCR [13-15]. Radiological findings are supportive but not necessary once tissue confirmation is made.

Our patient met criteria for probable mucormycosis upon presentation with a host factor of DKA, clinical findings of facial swelling and ophthalmoplegia, and imaging suggestive of sinus invasion and CST. Contrast-enhanced CT and MRI added to the radiological domain by demonstrating pansinusitis, periorbital gas, and CST. Later, histopathology revealed the diagnostic broad hyphae, further upgrading him to proven mucormycosis, but only after the fungus had already propagated to cause multifocal cerebral infarction.

Despite guideline therapy, including urgent and repeated sinus debridement procedures and a 10-day course of liposomal amphotericin B, our patient’s infection progressed to cavernous sinus involvement and multifocal cerebral infarction. These complications carry an extremely poor prognosis [3,6].

Early ROCM frequently masquerades as bacterial cellulitis or allergic sinus disease. As was the case with our patient, his facial swelling was initially attributed to an allergic reaction to azithromycin use. In a systematic review of 929 mucormycosis cases, delayed initiation of active antifungal therapy (>6 days) doubled mortality (82.9% vs. 48.6%) [17]. A subsequent study confined to hematologic malignancy found that every one-day delay in starting amphotericin B increased the odds of death by 11% [18]. Our patient’s initial diagnosis further emphasizes the diagnostic challenge of ROCM.

DKA further accelerates ROCM pathogenesis. Acidosis impairs neutrophil chemotaxis and function, while elevated serum glucose and iron promote unchecked fungal growth and tissue invasion, creating an environment uniquely permissive for *Mucorales* [5,19]. Prompt metabolic correction, therefore, represents a therapeutic as well as supportive priority.

Further management involves multiple debridement procedures. Debridement of necrotic tissue is indispensable because vascular thrombosis limits drug delivery. Guidelines recommend surgery within the first week of diagnosis and reiteration until viable margins are achieved [3]. In COVID-associated ROCM, debridement within seven days halved mortality compared with later surgery [7].

Orbital exenteration can be lifesaving in extensive orbital disease; several series report improved survival when performed before cerebral invasion [7]. Modern cohorts describe disease control with endoscopic decompression plus systemic and local amphotericin, avoiding the morbidity of exenteration [6]. Due to the patient’s CST and early infarcts, exenteration was unlikely to alter the outcome in this case; nevertheless, the case highlights the need for early discussion before cranial extension.

High-dose liposomal amphotericin B (≥5 mg/kg/day) remains first-line but is nephrotoxic, slow to sterilize tissue, and penetrates the CNS poorly. Prolonged therapy greater than six weeks is usually needed. The patient only completed a 10-day course as progressive CNS disease prompted transition to comfort care. Even with optimal therapy, overall mucormycosis mortality remains 40-50% and approaches 100% once the CNS is involved [3,6].

Isavuconazole and posaconazole provide oral and IV alternatives, with activity against *Mucorales*. In the phase III VITAL trial, isavuconazole produced 42-day survival comparable to amphotericin B with fewer adverse events [20]. In the study, adults greater than 18 years with proven or probable invasive mold disease, including 37 cases of mucormycosis based on EORTC/MSGERC criteria, who had received less than 48 hours of prior systemic antifungal therapy and were unsuitable for voriconazole or amphotericin B because of renal or other toxicity risks received intravenous or oral isavuconazole after a loading phase and could continue therapy for up to 180 days. In the mucormycosis subset, overall clinical/mycologic/radiologic response at end-of-treatment was 31%, and day-42 all-cause mortality was 38% [20].

Future improvements hinge on faster diagnostics, earlier intervention, and more effective systemic or targeted antifungal strategies.

## Conclusions

This case highlights the importance of early recognition of mucormycosis in high-risk patients presenting with facial swelling and DKA. Prompt diagnosis, aggressive surgical management, and antifungal therapy are paramount for survival. Clinicians should maintain a high index of suspicion, especially when imaging shows sinus involvement with orbital or cerebral extension. Further studies are warranted to assess the efficacy of newer antifungal agents.
